# Supplementary material for: The role of educational attainment and quality in U.S. regional variation in prevalence of dementia and CIND
Source: PLoS One. 2025 Sep 18;20(9):e0332410. doi: 10.1371/journal.pone.0332410 (PMC12445459; doi:10.1371/journal.pone.0332410)
Supplement: S1 Appendix — (DOCX) [file pone.0332410.s001.docx]

**S1 Appendix**

**S1 Figure.** Sample inclusion criteria and sample size after each selection criterion is applied


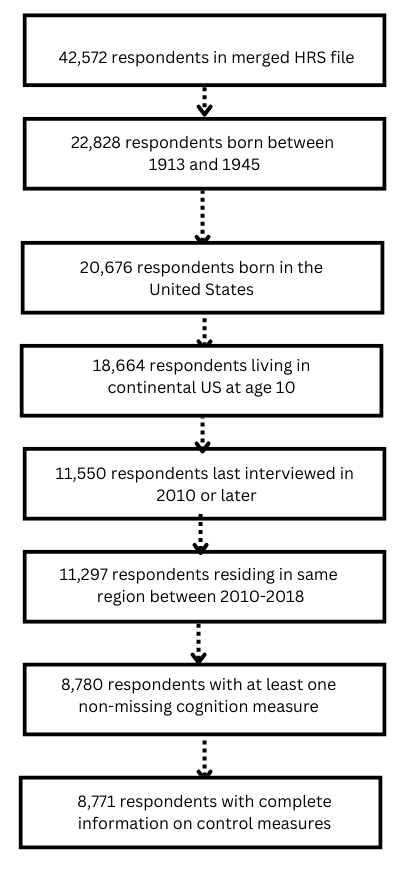


**S2 Figure**. Variation and levels of state-level education quality indicators used to construct the two state-level education quality factors by U.S. region from 1923 to 1955.


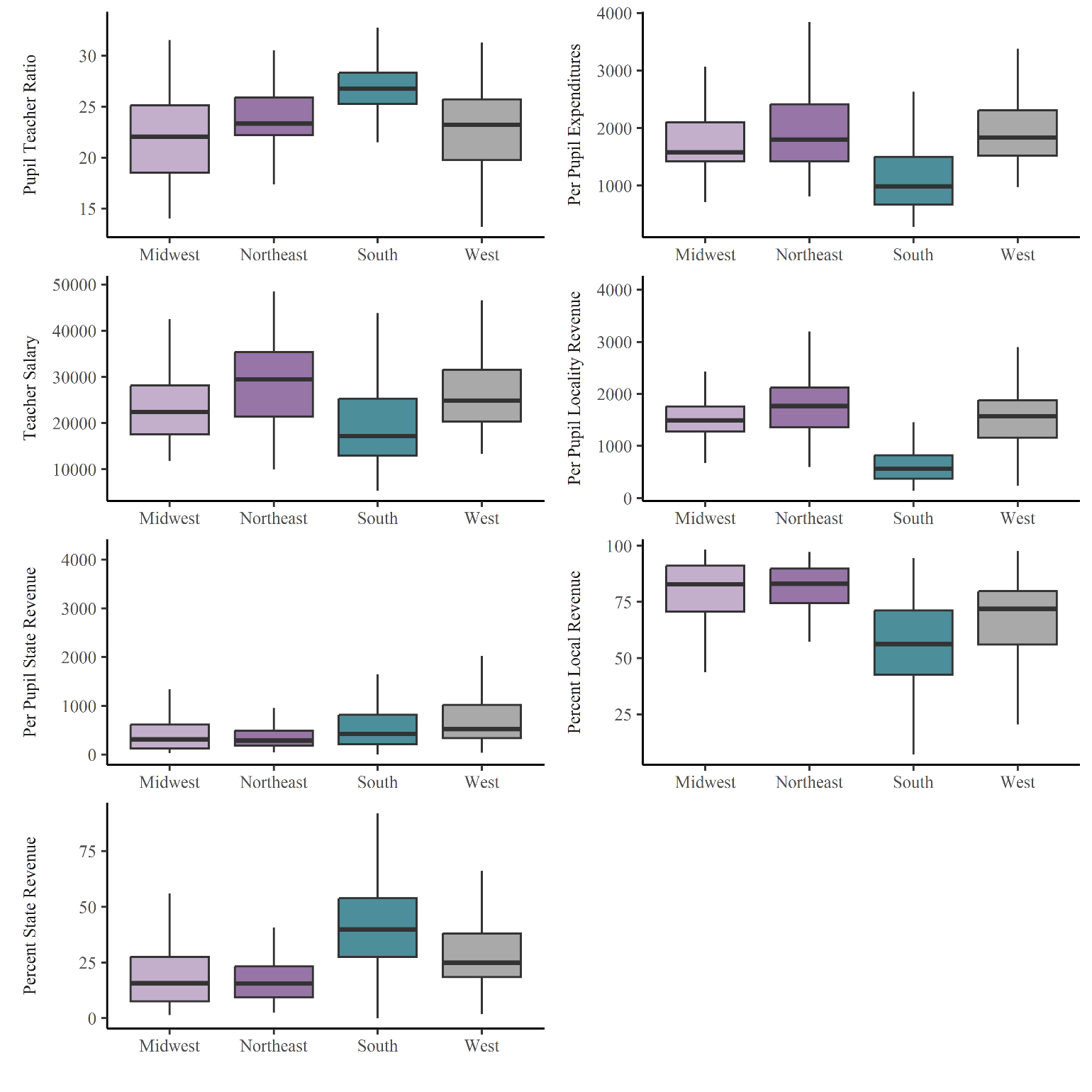


Notes: Values presented for Per Pupil Expenditures, Teacher Salary, Per Pupil Locality Revenue and Per Pupil State Revenue have been adjusted for inflation using 2021 Consumer Price Index Values.

**S3 Figure**: Eigenvalues of factors summarizing state-level education quality


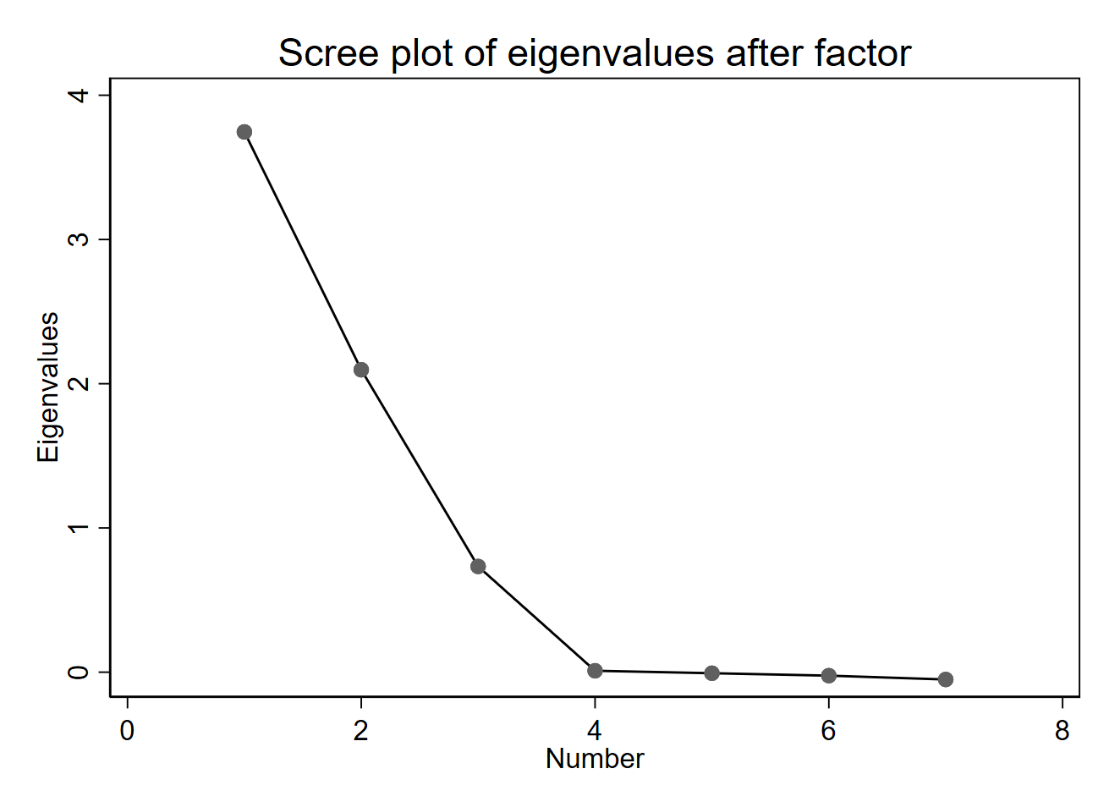


Data source: The 1919/20–1955Biennial Surveys of Education (BSE) of the United States

| **S1 Table:** Loadings of first four factors summarizing state-level education quality | | | | | |
| --- | --- | --- | --- | --- | --- |
| *Indicator* | Factor 1 | Factor 2 | Factor 3 | Factor 4 |  |
| Pupil teacher ratio | 0.23 | -0.12 | 0.87 | 0.00 |  |
| Per pupil expenditure ^a^ | -0.07 | 0.90 | -0.41 | 0.01 |  |
| Teaching salary ^a^ | 0.04 | 0.96 | 0.15 | 0.02 |  |
| Per pupil local revenue ^a^ | -0.57 | 0.71 | -0.34 | -0.05 |  |
| Per pupil state revenue ^a^ | 0.90 | 0.33 | -0.03 | 0.08 |  |
| % of revenue, local sources | -0.94 | 0.23 | -0.21 | 0.04 |  |
| % of revenue, state sources | 0.95 | -0.20 | 0.21 | -0.01 |  |
| ^a^ CPI adjusted to 2021 dollars | | | | | |

Data source: The 1919/20–1955 Biennial Surveys of Education (BSE) of the United States

**S2 Table**. Predicted probability of dementia or CIND by region among adults 65 and older, Health and Retirement Study 2010-2018.

| Region | Dementia (M1) | CIND  (M1) | Dementia (M2) | CIND  (M2) | Dementia (M3) | CIND  (M3) | Dementia (M4) | CIND  (M4) |
| --- | --- | --- | --- | --- | --- | --- | --- | --- |
| Northeast | 0.066 | 0.196 | 0.068 | 0.196 | 0.078 | 0.21 | 0.079 | 0.211 |
|  | (0.056,0.076) | (0.181,0.212) | (0.059,0.078) | (0.181,0.211) | (0.066,0.090) | (0.193,0.227) | (0.067,0.091) | (0.194,0.228) |
| Midwest | 0.071 | 0.194 | 0.07 | 0.189 | 0.072 | 0.192 | 0.073 | 0.193 |
|  | (0.063,0.079) | (0.182,0.206) | (0.063,0.077) | (0.177,0.200) | (0.064,0.080) | (0.181,0.204) | (0.065,0.081) | (0.181,0.204) |
| South | 0.094 | 0.204 | 0.088 | 0.199 | 0.08 | 0.188 | 0.079 | 0.188 |
|  | (0.087,0.101) | (0.194,0.214) | (0.081,0.095) | (0.190,0.209) | (0.073,0.086) | (0.179,0.198) | (0.072,0.085) | (0.178,0.198) |
| West | 0.07 | 0.182 | 0.082 | 0.199 | 0.087 | 0.205 | 0.087 | 0.204 |
|  | (0.060,0.080) | (0.168,0.195) | (0.070,0.093) | (0.185,0.214) | (0.075,0.100) | (0.190,0.220) | (0.075,0.099) | (0.190,0.219) |

Notes: Margins command used to predicted probability of dementia or CIND holding region characteristics at their observed values. 95% Confidence intervals shown in parentheses.

| **S3 Table**. Predicted difference in probability of dementia or CIND by state-level education resources and years of schooling among adults 65 and older, Health and Retirement Study 2010-2018. | | | | | | |
| --- | --- | --- | --- | --- | --- | --- |
|  | **Dementia** | | | **CIND** | | |
| Years of Schooling | ∆ High-Low Resources | 95% Confidence Interval | | ∆ High-Low Resources | 95 % Confidence Interval | |
| 8 | 0.077 | 0.049 | 0.105 | 0.068 | 0.033 | 0.103 |
| 9 | 0.060 | 0.039 | 0.080 | 0.067 | 0.037 | 0.097 |
| 10 | 0.044 | 0.029 | 0.059 | 0.062 | 0.037 | 0.086 |
| 11 | 0.031 | 0.020 | 0.043 | 0.053 | 0.033 | 0.073 |
| 12 | 0.021 | 0.012 | 0.030 | 0.043 | 0.026 | 0.059 |
| 13 | 0.013 | 0.006 | 0.020 | 0.033 | 0.018 | 0.047 |
| 14 | 0.007 | 0.001 | 0.013 | 0.023 | 0.009 | 0.037 |
| 15 | 0.003 | -0.002 | 0.009 | 0.015 | 0.000 | 0.029 |
| 16 | 0.001 | -0.004 | 0.006 | 0.008 | -0.007 | 0.022 |
| Notes: Margins command with pairwise comparison used to calculate difference in predicted probability of dementia or CIND at 8 to 16 years of schooling varying state-level education resources from low (-1) to high (1). All covariates set to their mean. Coefficients used in prediction taken from M4. Point estimates multiplied by 100 reflect the percentage point difference in the probability of having a cognitive status if resources were changed from low to high for an individual with a given years of schooling. | | | | | | |

| **S4 Table.** Relative risk ratios from multinomial regressions predicting cognitive status among adults 65 and older living in the South, Health and Retirement Study 2010-2018 | | | |
| --- | --- | --- | --- |
|  | M2 | M3 | M4 |
|  | RRR  (95% CI) | RRR  (95% CI) | RRR  (95% CI) |
| **Dementia (Ref.=Normal)** |  |  |  |
| Years of Schooling | 0.67*** | 0.68*** | 0.71*** |
|  | (0.64 , 0.69) | (0.66 , 0.71) | (0.68 , 0.75) |
| State-Level Income Inequality | 0.99 | 1.00 | 1.00 |
|  | (0.98 , 1.01) | (0.98 , 1.02) | (0.98 , 1.02) |
| State-Level Funding Source |  | 1.22* | 1.23* |
|  |  | (1.03 , 1.44) | (1.04 , 1.45) |
| State-Level Education Resources |  | 0.76*** | 0.75*** |
|  |  | (0.68 , 0.85) | (0.67 , 0.84) |
| Years of Schooling x Education Resources |  |  | 1.06** |
|  |  |  | (1.01 , 1.10) |
| **CIND (Ref.=Normal)** |  |  |  |
| Years of Schooling | 0.77*** | 0.78*** | 0.79*** |
|  | (0.75 , 0.79) | (0.76 , 0.81) | (0.76 , 0.81) |
| State-Level Income Inequality | 1.01 | 1.01 | 1.01 |
|  | (0.99 , 1.02) | (1.00 , 1.02) | (1.00 , 1.02) |
| State-Level Funding Source |  | 1.12* | 1.12* |
|  |  | (1.00 , 1.26) | (1.00 , 1.25) |
| State-Level Education Resources |  | 0.82*** | 0.80*** |
|  |  | (0.76 , 0.87) | (0.75 , 0.86) |
| Years of Schooling x Education Resources |  |  | 1.01 |
|  |  |  | (0.98 , 1.04) |
| Note: M2-M4 control for age, birth cohort, gender, and race/ethnicity. N=3,743 respondents contributing 14,188 observations. Standard errors clustered by respondent. Education centered at 12 years of schooling. Higher values on state-level education resources and state-level funding source reflect greater resources and greater state funding, respectively.  + p<.10, * p<.05, **p<.01 ***p<.001 | | | |

| **S5 Table.** Relative risk ratios from multinomial regressions predicting cognitive status among adults 65 and older living in the Northeast, Health and Retirement Study 2010-2018 | | | |
| --- | --- | --- | --- |
|  | M2 | M3 | M4 |
|  | RRR  (95% CI) | RRR  (95% CI) | RRR  (95% CI) |
| **Dementia (Ref.=Normal)** |  |  |  |
| Years of Schooling | 0.78*** | 0.78*** | 0.76*** |
|  | (0.72 , 0.86) | (0.72 , 0.86) | (0.69 , 0.85) |
| State-Level Income Inequality | 1.02 | 1.02 | 1.02 |
|  | (0.97 , 1.07) | (0.97 , 1.09) | (0.97 , 1.09) |
| State-Level Funding Source |  | 1.20 | 1.21 |
|  |  | (0.90 , 1.60) | (0.91 , 1.60) |
| State-Level Education Resources |  | 0.82* | 0.80* |
|  |  | (0.68 , 0.99) | (0.66 , 0.97) |
| Years of Schooling x Education Resources |  |  | 1.02 |
|  |  |  | (0.95 , 1.10) |
| **CIND (Ref.=Normal)** |  |  |  |
| Years of Schooling | 0.82*** | 0.82*** | 0.78*** |
|  | (0.78 , 0.86) | (0.78 , 0.86) | (0.73 , 0.84) |
| State-Level Income Inequality | 1.01 | 1.00 | 1.00 |
|  | (0.98 , 1.04) | (0.96 , 1.04) | (0.96 , 1.04) |
| State-Level Funding Source |  | 1.15 | 1.14 |
|  |  | (0.94 , 1.40) | (0.94 , 1.39) |
| State-Level Education Resources |  | 1.00 | 0.97 |
|  |  | (0.88 , 1.13) | (0.85 , 1.11) |
| Years of Schooling x Education Resources |  |  | 1.04+ |
|  |  |  | (0.99 , 1.10) |
| Note: M2-M4 control for age, birth cohort, gender, and race/ethnicity. N=1,219 respondents contributing 4,547 observations. Standard errors clustered by respondent. Education centered at 12 years of schooling. Higher values on state-level education resources and state-level funding source reflect greater resources and greater state funding, respectively.  + p<.10, * p<.05, **p<.01 ***p<.001 | | | |

| **Table S6.** Relative risk ratios from multinomial regressions predicting cognitive status among adults 65 and older living in the Midwest, Health and Retirement Study 2010-2018 | | | |
| --- | --- | --- | --- |
|  | M2 | M3 | M4 |
|  | RRR  (95% CI) | RRR  (95% CI) | RRR  (95% CI) |
| **Dementia (Ref.=Normal)** |  |  |  |
| Years of Schooling | 0.72*** | 0.72*** | 0.72*** |
|  | (0.67 , 0.77) | (0.67 , 0.78) | (0.67 , 0.77) |
| State-Level Income Inequality | 1.01 | 1.01 | 1.01 |
|  | (0.98 , 1.05) | (0.98 , 1.05) | (0.97 , 1.04) |
| State-Level Funding Source |  | 1.20+ | 1.19+ |
|  |  | (0.99 , 1.45) | (0.98 , 1.44) |
| State-Level Education Resources |  | 0.94 | 0.96 |
|  |  | (0.76 , 1.15) | (0.78 , 1.18) |
| Years of Schooling x Education Resources |  |  | 1.08* |
|  |  |  | (1.00 , 1.16) |
| **CIND (Ref.=Normal)** |  |  |  |
| Years of Schooling | 0.79*** | 0.79*** | 0.78*** |
|  | (0.76 , 0.82) | (0.76 , 0.82) | (0.75 , 0.82) |
| State-Level Income Inequality | 1.00 | 1.00 | 1.00 |
|  | (0.98 , 1.03) | (0.98 , 1.03) | (0.98 , 1.02) |
| State-Level Funding Source |  | 1.07 | 1.07 |
|  |  | (0.95 , 1.20) | (0.95 , 1.20) |
| State-Level Education Resources |  | 0.97 | 0.96 |
|  |  | (0.83 , 1.13) | (0.82 , 1.11) |
| Years of Schooling x Education Resources |  |  | 1.03 |
|  |  |  | (0.96 , 1.10) |
| Note: M2-M4 control for age, birth cohort, gender, and race/ethnicity. N=2,350 respondents contributing 8,907 observations. Standard errors clustered by respondent. Education centered at 12 years of schooling. Higher values on state-level education resources and state-level funding source reflect greater resources and greater state funding, respectively.  + p<.10, * p<.05, **p<.01 ***p<.001 | | | |

| **Table S7.** Relative risk ratios from multinomial regressions predicting cognitive status among adults 65 and older living in the West, Health and Retirement Study 2010-2018 | | | |
| --- | --- | --- | --- |
|  | M2 | M3 | M4 |
|  | RRR  (95% CI) | RRR  (95% CI) | RRR  (95% CI) |
| **Dementia (Ref.=Normal)** |  |  |  |
| Years of Schooling | 0.77*** | 0.77*** | 0.77*** |
|  | (0.72 , 0.82) | (0.72 , 0.82) | (0.71 , 0.83) |
| State-Level Income Inequality | 1.00 | 1.00 | 1.00 |
|  | (0.96 , 1.03) | (0.97 , 1.04) | (0.97 , 1.04) |
| State-Level Funding Source |  | 0.83* | 0.84+ |
|  |  | (0.69 , 1.00) | (0.69 , 1.00) |
| State-Level Education Resources |  | 0.95 | 0.93 |
|  |  | (0.75 , 1.20) | (0.73 , 1.18) |
| Years of Schooling x Education Resources |  |  | 0.99 |
|  |  |  | (0.93 , 1.07) |
| **CIND (Ref.=Normal)** |  |  |  |
| Years of Schooling | 0.83*** | 0.83*** | 0.82*** |
|  | (0.80 , 0.86) | (0.80 , 0.86) | (0.78 , 0.86) |
| State-Level Income Inequality | 0.99 | 0.99 | 0.99 |
|  | (0.97 , 1.01) | (0.97 , 1.01) | (0.97 , 1.01) |
| State-Level Funding Source |  | 0.98 | 0.98 |
|  |  | (0.87 , 1.10) | (0.87 , 1.10) |
| State-Level Education Resources |  | 1.02 | 1.01 |
|  |  | (0.89 , 1.17) | (0.87 , 1.17) |
| Years of Schooling x Education Resources |  |  | 1.01 |
|  |  |  | (0.97 , 1.06) |
| Note: M2-M4 control for age, birth cohort, gender, and race/ethnicity. N=1,459 respondents contributing 5,611 observations. Standard errors clustered by respondent. Education centered at 12 years of schooling. Higher values on state-level education resources and state-level funding source reflect greater resources and greater state funding, respectively.  + p<.10, * p<.05, **p<.01 ***p<.001 | | | |

| **S8 Table**. Estimates from KHB analysis decomposing changes in regression coefficients for U.S. South region of current residence by education and education quality measures stratifying on educational attainment | | | | |
| --- | --- | --- | --- | --- |
|  | **Dementia**  **≤ 12 years** | **CIND**  **≤12 years** | **Dementia**  **≥13 years** | **CIND**  **≥13 years** |
|  | b | b | b | b |
| Reduced South Region (ref=Midwest) ^a^ | 0.55*** | 0.25*** | 0.11 | 0.08 |
| Full South Region ^b^ | 0.08 | -0.06 | 0.01 | 0.03 |
| B_reduced_ – b_full_ | 0.47*** | 0.31*** | 0.10 | 0.05 |
| **% of South coefficient explained by:** | | |  |  |
| Years of Schooling | 49.5*** | 50.3*** | -49.3*** | -102.6*** |
| State-Level Funding Source | 16.4*** | 15.6*** | 50.5*** | 134.2*** |
| State-Level Education Resources | 34.2*** | 34.1*** | 98.8*** | 68.4*** |
|  |  |  |  |  |
| ^a^ Estimates from model adjusting for age, race, and gender.  ^b^ Estimates from model adjusting for age, race, gender, years of schooling, and state-level education quality indicators.  + p<.10, * p<.05, **p<.01 ***p<.001 | | | | |

| **S9 Table**. Relative risk ratios from multinomial regressions predicting cognitive status among adults 65 and older, residing in the same region at birth, age 10, and time of interview, Health and Retirement Study 2010-2018. | | | | | |
| --- | --- | --- | --- | --- | --- |
|  | M1 | M2 | M3 | M4 | |
|  | RRR  (95% CI) | RRR  (95% CI) | RRR  (95% CI) | RRR  (95% CI) | |
| **Dementia (Ref = Normal)** |  |  |  |  | |
| Region at Interview (Ref = Midwest) | 1.00 | 1.07 | 1.26 | 1.24 |  |
| Northeast | (0.77 , 1.30) | (0.81 , 1.41) | (0.92 , 1.71) | (0.91 , 1.69) | |
|  | 2.02*** | 1.75*** | 1.27 | 1.26 | |
| South | (1.63 , 2.52) | (1.39 , 2.20) | (0.92 , 1.76) | (0.91 , 1.74) | |
|  | 1.11 | 1.64** | 1.74*** | 1.69** | |
| West | (0.82 , 1.51) | (1.20 , 2.23) | (1.26 , 2.41) | (1.22 , 2.33) | |
|  |  | 0.70*** | 0.70*** | 0.71*** | |
| Years of Schooling |  | (0.68 , 0.73) | (0.68 , 0.73) | (0.69 , 0.74) | |
|  |  | 1.00 | 1.01 | 1.01 | |
| State-Level Income Inequality |  | (0.99 , 1.02) | (0.99 , 1.02) | (0.99 , 1.02) | |
|  |  |  | 1.09 | 1.08 | |
| State-Level Funding Source |  |  | (0.96 , 1.24) | (0.95 , 1.23) | |
|  |  |  | 0.83* | 0.84* | |
| State-Level Education Resources |  |  | (0.72 , 0.96) | (0.72 , 0.97) | |
|  |  |  |  | 1.04* | |
| Years of Schooling x Education Resources |  |  |  | (1.00 , 1.07) | |
| **CIND (Ref = Normal)** |  |  |  |  | |
| Region at Interview (Ref = Midwest) | 1.04 | 1.07 | 1.17+ | 1.17+ | |
| Northeast | (0.89 , 1.22) | (0.91 , 1.26) | (0.98 , 1.40) | (0.98 , 1.40) | |
|  | 1.43*** | 1.35*** | 1.06 | 1.06 | |
| South | (1.25 , 1.65) | (1.18 , 1.55) | (0.86 , 1.31) | (0.86 , 1.30) | |
|  | 0.91 | 1.16 | 1.16 | 1.15 | |
| West | (0.75 , 1.10) | (0.96 , 1.41) | (0.95 , 1.42) | (0.94 , 1.40) | |
|  |  | 0.79*** | 0.79*** | 0.79*** | |
| Years of Schooling |  | (0.77 , 0.81) | (0.77 , 0.81) | (0.78 , 0.81) | |
|  |  | 1.01 | 1.01 | 1.01 | |
| State-Level Income Inequality |  | (1.00 , 1.02) | (1.00 , 1.02) | (1.00 , 1.02) | |
|  |  |  | 1.12** | 1.12** | |
| State-Level Funding Source |  |  | (1.03 , 1.22) | (1.03 , 1.22) | |
|  |  |  | 0.90* | 0.89** | |
| State-Level Education Resources |  |  | (0.82 , 0.98) | (0.81 , 0.97) | |
|  |  |  |  | 1.02 | |
| Years of Schooling x Education Resources |  |  |  | (1.00 , 1.04) | |
| Note: M1-M4 control for age, birth cohort, gender, and race/ethnicity. N=6,123 respondents contributing 23,276 observations. Standard errors clustered by respondent. Education centered at 12 years of schooling. Higher values on state-level education resources and state-level funding source factors reflect greater state-level resources for public schools and greater state funding of public schools, respectively.  + p<.10, * p<.05, **p<.01 ***p<.001 | | | | | |
